# AUV Path Planning Considering Ocean Current Disturbance Based on Cloud Desktop Technology

**DOI:** 10.3390/s23177510

**Published:** 2023-08-29

**Authors:** Siyuan Hu, Shuai Xiao, Jiachen Yang, Zuochen Zhang, Kunyu Zhang, Yong Zhu, Yubo Zhang

**Affiliations:** 1 School of Futrue Technology, Tianjin University, Tianjin 300072, China; husiyuan77@tju.edu.cn; 2 School of Electrical and Information Engineering, Tianjin University, Tianjin 300072, China; yangjiachen@tju.edu.cn (J.Y.); yongzhu@tju.edu.cn (Y.Z.); 3 Tianjin Zhuo Lang Technology Development Co., Ltd., Tianjin 300131, China; zhangzuochen@troila.com (Z.Z.); zhangkunyu@troila.com (K.Z.); 4 Tianjin Institute of Software Engineering, Tianjin 300387, China; ybzhang@tjise.edu.cn; 5 School of Software, Tiangong University, Tianjin 300387, China

**Keywords:** deep learning, path planning, ocean data, ocean current, cloud desktop

## Abstract

In the field of ocean energy detection, Autonomous Underwater Vehicles (AUVs) offer significant advantages in terms of manpower, resource, and energy efficiency. However, the unpredictable nature of the ocean environment, particularly the real-time changes in ocean currents, poses navigational risks for AUVs. Therefore, effective path planning in dynamic environments is crucial for AUVs to perform specific tasks. This paper addresses the static path planning problem and proposes a model called the noise net double DQN network with prioritized experience replay (N-DDQNP). The N-DDQNP model combines a noise network and a prioritized experience replay mechanism to address the limited exploration and slow convergence speed issues of the DQN algorithm, which are caused by the greedy strategy and uniform sampling mechanism. The proposed approach involves constructing a double DQN network with a priority experience replay and an exploration mechanism using the noise network. Second, a compound reward function is formulated to take into account ocean current, distance, and safety factors, ensuring prompt feedback during the training process. Regarding the ocean current, the reward function is designed based on the angle between the current direction and the AUV’s heading direction, considering its impact on the AUV’s speed. As for the distance factor, the reward is determined by the Euclidean distance between the current position and the target point. Furthermore, the safety factor considers whether the AUV may collide with obstacles. By incorporating these three factors, the compound reward function is established. To evaluate the performance of the N-DDQNP model, experiments were conducted using real ocean data in various complex ocean environments. The results demonstrate that the path planning time of the N-DDQNP model outperforms other algorithms in different ocean current scenarios and obstacle environments. Furthermore, a user console-AUV connection has been established using spice cloud desktop technology. The cloud desktop architecture enables intuitive observation of the AUV’s navigation posture and the surrounding marine environment, facilitating safer and more efficient underwater exploration and marine resource detection tasks.

## 1. Introduction

With the progress and development of science and technology [1], there is an increasing demand for resources. As land resources become increasingly exploited, attention has turned toward the ocean for the detection of marine resources. The ocean covers approximately 70% of the Earth’s surface, providing a vast potential for the harvesting of ocean energies. However, due to the challenging ocean environments, it has become crucial to explore ways to harvest ocean energies in a reasonable and convenient manner. Detecting ocean energies is an essential step in this process, as it allows researchers to determine the types and quantities of ocean energies in advance, enabling proper resource allocation for harvesting. Unlike resource exploration on land, ocean exploration presents uncertain factors and numerous unknown risks. Elements such as reefs, currents, and whirlpools can pose dangers to ships and crew. To mitigate the damage caused by unpredictable risks, the use of Autonomous Underwater Vehicles (AUVs) for ocean exploration and energy detection has become a growing trend in this field. Effective path planning in dynamic ocean environments, considering real-time factors such as ocean currents, is crucial for the safe and energy-efficient navigation of AUVs. Path planning is a typical optimization problem and holds practical value as a foundational technology for AUVs. Consequently, the underwater path planning problem has garnered increasing attention. However, underwater path planning is challenging and uncertain due to various factors, such as ocean currents and obstacles. Optimizing the path involves establishing objectives and traversing states. Reinforcement learning, a model of continuous decision-making, is capable of outputting optimal actions based on the current state and has found wide applications in decision-making problems, such as interactive games and game theory. In recent years, reinforcement learning has emerged as a hot topic in path planning research due to its ability to learn in complex environments. Previous research methodologies have demonstrated the feasibility and effectiveness of reinforcement learning algorithms in path planning, yielding positive results. However, challenges remain in applying reinforcement learning to ocean path planning:

(1) Most existing methods either neglect the influence of ocean currents on AUV positions or only account for single-directional simulated currents.

(2) The current random exploration strategies prove inadequate in thoroughly exploring complex environments, resulting in an insufficient number of effective samples during the training phase and a higher likelihood of becoming trapped in local optimum solutions.

(3) During the training phase, uniform sampling is unsuitable for targeted learning concerning samples with varying prediction errors.

Hence, it is essential to consider the impact of ocean currents on AUV positions and the design of the reward function, thereby incorporating this factor into the input state of the reinforcement learning algorithm. Moreover, the exploration strategy is parameterized to allow autonomous adjustment of the extent to which relevant noise is introduced. Additionally, prioritization of samples based on prediction errors is implemented, facilitating non-uniform sampling and enhancing the network’s convergence speed and stability.

Furthermore, during each test after training the optimal model, it is necessary for the AUV to be manually transported back to the starting point from the endpoint. This process consumes significant manpower and resources. Additionally, the underwater working environment of AUVs restricts the ability to observe the AUV’s navigation posture and the marine environment during navigation, posing challenges to the exploration of marine environments and the development of subsequent tasks.

To address these issues, the Cloud Desktop Transfer Protocol is introduced, which enables the transmission of graphical desktop screen, audio, and video information from a remote server’s virtual computer to the client. This facilitates interaction with external devices connected to the client. By leveraging cloud desktop technology, remote control of AUVs becomes feasible, consequently reducing the operational manpower and resource requirements. Furthermore, the cloud desktop technology allows for the visualization of the marine environment exploration and subsequent detection of AUV navigation posture and real-time marine conditions, significantly enhancing the efficiency of ocean exploration.

To overcome the inadequate exploration and slow convergence of the original DQN algorithm in complex environments, this paper proposes a novel model called the Noise Network and Priority Experience Replay Dual DQN Network (N-DDQNP). It combines the noise network and priority experience replay mechanisms. The key contributions of this research are summarized as follows:

(1) The N-DDQN model is proposed by integrating the noise network with the DQN model, thereby enhancing the model’s exploration capability and addressing the exploration-utilization imbalance issue.

(2) To overcome the slow training speed caused by random sampling, this paper introduces the priority experience replay mechanism into the N-DDQN model, allowing for non-uniform sampling based on sample priority. This approach effectively resolves the slow convergence problem associated with random sampling.

(3) A compound reward function is devised to ensure the stability of the training process of the N-DDQNP model in a two-dimensional real current environment, as well as to prioritize the safety and time efficiency of the planning path. When the distance between the target point and the next position is shorter than the distance between the target point and the current position, a positive reward is assigned, thereby favoring smoother paths as a superior choice. In terms of rewards related to the ocean currents, they are determined by the ratio of the actual velocity to the AUV’s velocity. This captures the influence of the ocean currents on the AUV’s movement by considering the actual velocity. Regarding the safety factor, a significant penalty is imposed when the AUV collides with obstacles. This paper comprehensively incorporates the aforementioned three factors to formulate a compound reward function.

(4) A connection between the user’s control center and the AUV is established using Spice cloud desktop technology. On one hand, cloud desktop technology enables remote control of AUVs, simplifying and streamlining their operation. On the other hand, high-quality transmission images provided by the cloud desktop technology allow users to clearly observe the AUV’s navigation posture and environment. This enables easy data collection and facilitates improvements and extensions to subsequent experiments. Furthermore, to address the instability of cloud desktop control technology in the presence of network fluctuations, a predefined return path for the AUV and asynchronous data uploading functionality have been implemented. This ensures the ability to observe the AUV’s marine environment and control the AUV, even in the presence of network fluctuations.

Path planning algorithms can be categorized into traditional search or random sampling-based algorithms, as well as neural network-based algorithms that possess learning capabilities [2]. Among the traditional algorithms, Dijkstra’s algorithm, A-star (A*), Artificial Potential Field (APF), and Rapidly-exploring Random Trees (RRT) hold certain applicability in the marine environment [3]. Dijkstra’s algorithm adopts a breadth-first search approach, expanding from the initial point and recording the shortest distance from the initial point to each vertex until the destination is reached. However, using Dijkstra’s algorithm necessitates representing the environment as a directed graph [4]. Search efficiency tends to decrease when the number of nodes becomes large. Kirsanov et al. [5] applied an enhanced version of Dijkstra’s algorithm to solve the AUV path planning problem in a two-dimensional plane. However, since the ocean current environment exhibits unidirectional characteristics, it is challenging to justify its applicability in real environments. A* algorithm extends Dijkstra’s algorithm by employing a heuristic function to calculate path cost and continually reduce the search space. This method proves effective for solving the shortest path problem in static grid maps. However, as the search scope increases, the time complexity of the method rises, leading to longer path calculation times. Rao et al. [6] proposed an approach for determining energy-optimal paths accounting for the influence of ocean currents. Nonetheless, it lacks obstacle avoidance capabilities, thereby compromising safety. OCi-RRT (Ocean current improved RRT) [7] is a path planning solution that considers the impact of ocean currents. Nevertheless, the experimental environment remains limited to two dimensions, resulting in low sampling complexity.

The APF algorithm assumes the existence of virtual space. In the force field, the robot is attracted by the target and repelled by obstacles to prevent a collision. The operation of the APF algorithm requires the establishment of a potential field with complete environmental information. It has the advantages of intuitiveness and low computation [8]. Jiang et al. adopted the APF algorithm for obstacle avoidance and path planning of a 6 DOF robot in the workspace [8]. Fan et al. proposed an improved APF method for path planning in static and dynamic environments, enabling an AUV to make obstacle avoidance decisions based on its own state and the movement of obstacles [9]. Wu et al. proposed a discrete fast search random tree (DRRT) based on the RRT algorithm [10].

Simulation results demonstrate that the DRRT algorithm has the advantage of dealing with dynamic environments but lacks consideration of the influence of ocean currents [11]. Thus, its effectiveness in current environments cannot be demonstrated. To address the inefficiency of the RRT algorithm in search, Gammell et al. utilized elliptic equations to solve the local path planning problem [12]. They proposed an improved fast search algorithm called IRRT* that employs an ellipse-based heuristic method to limit the search scope. However, applying the elliptic equation in current environments increases calculation complexity, and the presence of currents may cause position offsets [13]. The restricted sampling points limit the algorithm’s adaptability. Cheng et al. introduced the APF algorithm into the heuristic function of bidirectional RRT [14] to solve the problem of dynamic barriers. This approach combines global navigation and local path planning, thereby improving the expansion efficiency of random trees [15]. Sharma et al. utilized an improved A* algorithm with circular boundaries to ensure the effectiveness of the experimental environment in the presence of static and moving obstacles and changes in ocean currents [16]. However, the lack of consideration for obstacles reduces the applicability of the experiment [17]. Furthermore, this method lacks verification. Xy et al. proposed an interval optimization scheme [18] to plan the time-optimal path of an AUV in a marine environment with dynamic and uncertain flow fields.

With the continuous innovation of deep learning, deep learning methods have gained attention in autonomous underwater vehicle (AUV) path planning due to their strong ability to learn data features and model complex environments. Among them, reinforcement learning (RL) has become a focus due to its unsupervised characteristics and self-learning ability. Bhopa et al. used RL methods to directly learn control strategies from high-dimensional perceptual inputs [19]. Nie et al. proposed a path planning algorithm based on depth Q network for the simulation environment of mobile robots [20]. Duan et al. combined potential energy fields with Q-learning [21], initializing the Q-value with potential energy value as search information to guide the mobile robot in rapid convergence. However, the expansion of state space makes Q-learning difficult to converge. Due to its good path-planning ability in unknown environments, RL algorithms have been applied to ocean robot systems. Chen et al. proposed a path planning and control method based on Q-learning for steering cargo ships without any human experience input [22]. Zhao et al. proposed a new RL strategy [23] to solve the optimal stability problem of unknown nonlinear systems with uncertain input constraints. The traditional RL algorithm [6] is one of the effective means for solving local path planning. However, it faces challenges such as insufficient computing resources when the environment becomes more complex. The environmental state used in this article includes information such as the location of obstacles and the direction of ocean currents, requiring substantial computing power for effective training [24].

With the rapid development of virtualization technology, systems like WEBOS systems and Virtual Desktop Infrastructure (VDI) systems have emerged. The primary objective of these systems is to address various issues encountered in the use of traditional desktops, making desktop usage more efficient and user-friendly [25]. Currently, VDI is the mainstream architecture and deployment method for desktop cloud solutions, resolving issues associated with traditional desktops such as management, security, and energy conservation [26]. Originally developed by Qumranet, Spice functions as a remote desktop display protocol for connecting with virtual machines. The Spice framework, built upon the Spice protocol, encompasses a client, a server, and associated components. The open-source Spice protocol client employs the GTK framework to meet cross-platform requirements [27]. The open-source Spice server serves the QEMU process and operates on the cloud desktop host machine. In recent years, high-quality image transmission technology has developed rapidly [28], providing the possibility of real-time transmission of AUV’s navigation posture and surrounding marine environment through cloud desktop technology. The advantages of high dynamic range (HDR) technology [29] in scene reproduction can also make the underwater environment rendered more realistic. Miu-Ling Lam [30] presents the design and implementation details of a novel cloud-based system architecture for robotic path planning. The suggested solution, referred to as PPaaS (Path Planning as a Service), utilizes a hierarchical architectural configuration consisting of three layers: the cloud server layer, the cloud engine layer, and the client robot layer. The Hadoop Distributed File System is employed to implement the cloud server layer.

## 2. Method

**Figure 1:**
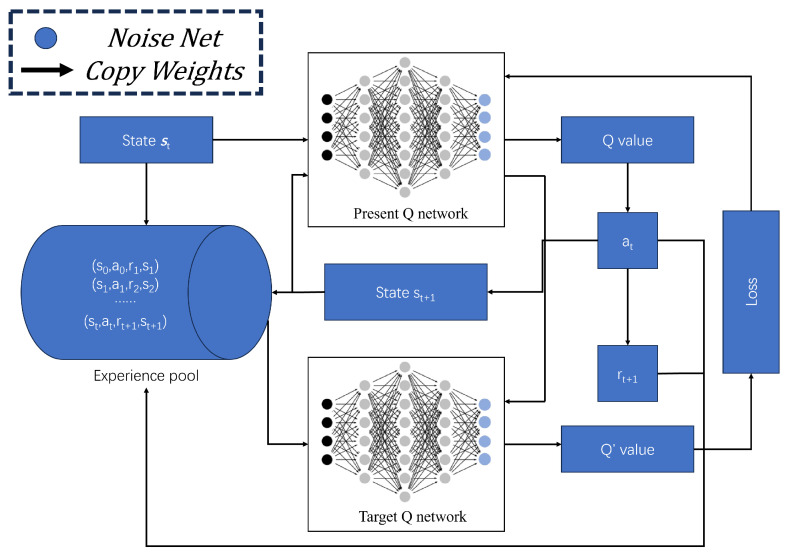
N-DDQNP Framework.

This section presents a static path planning model for underwater AUVs based on N-DDQNP. The model addresses the limitations of reinforcement learning models based on DQN, such as incomplete exploration using greedy strategies and slow convergence due to uniform sampling. The overall framework of the N-DDQNP model is depicted in Figure 1. The N-DDQNP model builds upon the DDQN framework and incorporates parameterized noise as an exploration strategy. This ensures that the output actions during the initial training phase exhibit a certain level of randomness, thus preventing local optimization issues resulting from inadequate exploration. The network parameters are updated using the gradient descent method, allowing for adaptive adjustment of the noise level. Additionally, the model incorporates a priority experience replay mechanism, where the prediction error serves as the priority value index for samples, and non-uniform sampling is performed accordingly. In the N-DDQNP model, the output layer is represented by the Noise Net, and the selection of actions is based on the Q-value (Q(s,a)). After executing an action, the agent receives rewards and the next state from the environment. Samples collected during the exploration process are stored in an experience pool, and training samples are extracted using the priority sampling mechanism.

### 2.1. DDQN Model

This section employs a value function-based reinforcement learning algorithm for making action decisions. Deep Reinforcement Learning (DRL) combines deep neural networks with RL techniques, allowing the neural network output to replace the Q-value output, effectively addressing high-dimensional disaster problems [7]. DDQN, as a DRL method, resolves the issue of overestimating Q-values that existed in the DQN network. To obtain the output for state $s_{t+1}$, we compute the action $a^{*}$ corresponding to the maximum Q-value in the current Q network.

(1) $$a^{*}=\arg\max_{a}Q\left(s_{t+1},a\;;\omega \right)$$

In the target Q network, when the input state is $s_{t+1}$, action $a^{*}$ is the corresponding Q-value. Therefore, the label of the current Q network is:

(2) $$y=r_{t+1}+\gamma Q^{\prime }\left(s_{t+1},\arg\max_{a}Q\left(s_{t+1},a\;;\omega \right);\omega^{\prime }\right)$$

Compared with the DQN algorithm, its constructed estimation target directly selects the largest Q-value in the target network, ignoring the relationship with the current network, which will lead to overestimation. According to the MSE method, the loss function for calculating DDQN is Formula (3), and the parameters are updated by the gradient descent method according to Formula (4).

(3) $$L\left(\omega \right)={\left(y-Q\left(s_{t},a\;;\omega \right)\right)}^{2}$$

(4) $$\omega =\omega -\alpha \frac{dL(\omega )}{d\omega }$$

To maintain the stability of the prediction target over a certain time period, the target network copies the current network parameters ω every X episodes for updating purposes. Consequently, a larger update interval of the target network [31] ensures a more stable algorithm, albeit at the cost of reduced update frequency and convergence speed. Therefore, it is crucial to establish an appropriate update interval. This parameter replacement method, where the network parameters are entirely replaced, is referred to as a “hard update” and is a commonly employed update method in enhanced DQN algorithms.

### 2.2. Noise-Net

The original DQN algorithm typically employs the ϵ-greedy strategy for random exploration. Since the current network directly outputs the predicted values for each action, selecting the action with the highest value directly would cause the network to lose its exploration capability. Therefore, the greedy strategy is usually used to strike a balance between exploration and exploitation: the optimal action is output with a probability of 1−ϵ, while a random action is chosen with a probability of ϵ, ensuring a certain level of randomness in exploration and diversity in the experience pool. To ensure network convergence during the update process, the value of ϵ gradually decays to guarantee the output of the optimal action [18]. The decay mode of ϵ affects the network’s exploration capability. If the decay rate is too fast, it may result in insufficient exploration. Conversely, if the decay rate is too slow, it may lead to slow convergence and excessive randomness in the output action. Therefore, this section employs a noise network instead of the greedy strategy. The noise network leverages the perturbation of learned network weights to drive exploration. Unlike the greedy strategy that adds irrelevant perturbations at each step, the noise network parameters remain stable between two updates, ensuring that changes in the noise network weights consistently impact subsequent multi-step decision-making. The perturbation of the noise network is sampled from a noise distribution, and the variance parameters are learned through the gradient descent process of the reinforcement learning loss function.

The noise network has two implementations [32]: independent Gaussian noise using independent noise for each weight and decomposed Gaussian noise using different independent noise for input and output. Independent Gaussian noise samples *w*, bas independent Gaussian distributions. It needs to sample from p×q+q distributions. To decompose the Gaussian noise, first sample *p* units of Gauss to form a p×1-dimensional matrix, and then generate a q×1-dimensional matrix in the same way. Use the matrix product method to generate a p×q-dimensional matrix. A total of p+q Gaussian distributions need to be sampled, reducing the sampling overhead.

To reduce the time of generating random numbers in the algorithm, this section adopts the decomposition of Gaussian noise [33]. The noise network is a parametric neural network, and its weight and deviation are disturbed by the noise parameter function. The network parameters are adaptively adjusted according to the gradient descent. The forward calculation formula of the full connection layer is y=wx+b, where $x\in R^{p}$ is the network input, $w\in R^{q\times p}$ is the network weight, and $b\in R^{q}$ is the offset term. The corresponding noise full connection layer is:

(5) $$y\overset{\;\mathrm{def}\;}{=}\left(\mu^{w}+\sigma^{w}⊙\varepsilon^{w}\right)x+\mu^{b}+\sigma^{b}⊙\varepsilon^{b}$$

In this section, the N-DDQNP model is proposed, which replaces the fully connected layer of the last layer with a noise network layer. Additionally, the greedy strategy is discarded, empowering the noise network to independently determine the amount of noise to introduce during the training process.

### 2.3. Experience Playback Mechanism

The traditional DQN algorithm employs a uniform random sampling approach for the experience replay mechanism [33]. In this mechanism, all samples in the experience pool are assigned equal importance, and a fixed number of samples are selected for network training each time. When the number of samples in the experience pool reaches its maximum capacity, older experiences overwrite the earliest ones. This experience replay mechanism breaks the correlation between training samples, mitigates overfitting during gradient updates, and reduces variance.

Although the experience replay method ensures that all samples have equal opportunities to be sampled, in reality, each sample contributes differently to network training. Samples with large disparities between their predicted values and true values require more attention and learning, therefore necessitating a non-uniform sampling strategy. The key factor in determining priority is the TD error value, as indicated in the formula, with *y* representing the label of the current network Q-value [20].

(6) $$\delta_{j}=y-Q\left(s_{t},a\;;\omega_{t}\right)$$

The sampling probability of the sample given priority proportion is the formula, where $P_{j}$ is the priority value, specifically $\left|\delta_{j}\right|+\chi$, χ are very small values. It is guaranteed that samples with very small priority values can also be sampled α is the priority adjustment parameter, which controls the degree of priority sampling. When α is 0, it is uniform random sampling.

(7) $$P\left(j\right)=\frac{P_{j}^{\alpha }}{\sum_{i}P_{i}^{\alpha }},\alpha \in \left[0,1\right]$$

However, the introduction of priority sampling leads to a change in the original sample probability distribution, which will lead to the inaccuracy of the model. Therefore, the weight of samples should also be used for error correction when updating. The calculation method is shown in the formula, where *N* is the capacity of the experience pool, $\varpi_{L}$ is the maximum weight, and β is the degree of correction, which is limited to [0,1]. When β = 0, no correction is made β value increases to 1 with the training process, and its calculation method is shown in the formula, where $\beta_{\mathrm{initial}}$ = 0.4, $\omega_{\beta }$ = 2000, *k* is the current training rounds.

(8) $$\varpi_{j}=\frac{1}{{\left(NP_{j}\right)}^{\beta }\max\varpi_{l}}$$

(9) $$\beta =\min\left(1,\beta_{\mathrm{initial}\;}+\frac{k\left(1-\beta_{\mathrm{initial}\;}\right)}{\omega_{\beta }}\right)$$

### 2.4. Reward Function

The convergence of a reinforcement learning algorithm hinges on the reasonability of its reward function design. In scenarios with complex state and action spaces, achieving goals within a limited number of steps becomes challenging when relying on sparse rewards. To tackle this issue in the context of ocean current path planning, this section presents a universally applicable reward function. This function incorporates factors such as target distance, current utilization, and a factor of safety.

The fixed reward is set to 50 when the AUV reaches the target location. It is important to avoid setting the target reward excessively high, as this would diminish the penalty effect when encountering obstacles. When the target has not been reached, this paper utilizes the Euclidean distance between the endpoint and the current position, as well as the subsequent forward position, to evaluate the action. The distance from the current position to the target is denoted as $\xi_{t}$. $R_{d}$ represents the distance reward:

(10) $$R_{d}=\arctan k_{1}\left(\xi_{t}-\xi_{t+1}-\delta_{d}\right)$$

The arctangent function is used to limit the range of distance reward, and $k_{1}$ is the coefficient to adjust the reward range, with $\delta_{d}$ as the benchmark, which can reduce the occurrence of zigzag paths [20]. $\delta_{d}$ is set to the distance per unit time AUV in still water.

The rewards related to the ocean current are determined by the actual speed $\left|\vec{v_{r}}\right|$ and AUV speed $\left|\vec{v_{b}}\right|$ ratio setting. The actual speed of the AUV will be greater than its basic speed when it moves along the ocean current

(11) $$R_{c}=\arctan k_{2}\left(\frac{\left|\vec{v_{r}}\right|}{\varepsilon_{2}}-1\right)$$

(12) $$\varepsilon_{2}=\left|\vec{v_{b}}\right|+\delta_{c}$$

(13) $$\delta_{c}=\tau_{c}\left|\vec{v_{c}}\right|.$$

In the event of a collision between the AUV and an obstacle, a fixed penalty of −50 is assigned, thereby conveying the imperative nature of avoiding collisions. Setting a small penalty value is not conducive to encouraging the AUV to take effective obstacle-avoidance actions. Conversely, setting the penalty value too high may result in an overly conservative strategy, leading to an increase in navigation distance. Therefore, the collision reward function is defined as follows:

(14) $$R_{o}=-50$$

The final formula is as follows. $\lambda_{d}$, $\lambda_{c}$ represent the ratio of $R_{d}$, $R_{c}$.

(15) $$R=\lambda_{d}R_{d}+\lambda_{c}R_{c}+R_{o}$$

### 2.5. Cloud Desktop Architecture

**Figure 2:**
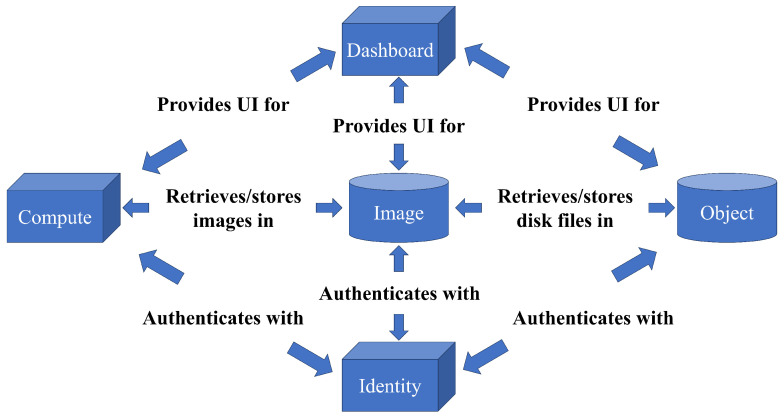
Diagram of the relationship between OpenStack components.

The SPICE client component is deployed on the endpoint device, which can be a client or a dedicated console, responsible for displaying each virtual desktop. The SPICE server is integrated with the KVM virtual machines as a dynamic connection library and communicates with the clients using the SPICE protocol [34]. On the server side, the QXL driver is deployed within the virtual machines that provide virtual desktop services. Its role is to receive graphics commands from the operating system and applications and convert them into QXL graphics device commands for KVM. Additionally, the QXL device is deployed within the virtualized hypervisor of the KVM server to handle graphics operations from each virtual machine. The SPICE protocol facilitates communication between the client and server through various dedicated channels, with each channel assigned to a specific type of data transmission and communication. The content of each channel can be transmitted through the corresponding graphics command data stream. The SPICE protocol ensures that each channel is dedicated to a specific type of data transfer and communication [35]. By utilizing the SPICE protocol, users can experience the same functionality as a real PC, allowing playback of videos, audio, and more directly within the virtual machine. To establish a robust cloud platform, OpenStack is chosen among the current mainstream cloud computing platforms Eucalyptus, OpenNebula, OpenStack, and CloudStack. OpenStack provides comprehensive performance and serves as the foundation for the server side of the cloud desktop’s basic services. The OpenStack platform consists primarily of five components: Nova, Swift, Glance, Keystone, and Horizon, with their interdependencies illustrated in Figure 2. In this experiment, remote observation and control of the AUV were achieved by deploying the OpenStack platform. To enable observation of the marine environment during the AUV’s navigation and overcome the challenges of remote data transmission in cloud desktops, the Swift component of OpenStack was pre-deployed within the AUV. This facilitated the storage of observed data of the surrounding marine environment during the AUV’s navigation, which was periodically transferred to the Horizon component of the platform for observation and display. This allowed for delayed observation of the marine environment. In terms of control, this study utilized cloud desktop technology and the OpenStack platform to control the AUV’s return from the endpoint to the starting point, reducing the costs associated with manually resetting the AUV’s position during multiple experiments. To address issues such as network latency and interruptions in cloud desktop control, a pre-set return path was established before conducting the AUV submersion experiment. The AUV’s position and status were periodically reported to the control console. If abnormal status indications were received, the return path was re-imported to the AUV via the OpenStack platform to ensure its safe return. This pre-set return path also mitigated the risk of AUV disconnection in the event of network link interruptions.

## 3. Experiment Results

In this section, two different ocean current environments were selected for experimentation. They encompass the regions of 123.12° E–127.23° E and 28.78° N–32.12° N, with a depth of 150 m. The ocean current data for January and December 2018 were obtained, denoted as environments C1 and C2, respectively. These data were sourced from the National Oceanic Data Center and were obtained using the Princeton Ocean Model with a generalized coordinate system (POMgcs) as the oceanic dynamical model, with an original horizontal resolution of 1/2°. The assimilation of ocean data was performed using a multi-grid three-dimensional variational ocean data assimilation method. The data are stored in the NetCDF format, which is a standardized format for information definition and encoding, specifically designed for array-oriented data and suitable for network sharing. NetCDF enables efficient storage, processing, collection, and distribution of network data. Its versatility in handling large array-oriented data has led to its wide application in various fields, including oceanography and environmental modeling.

The experimental environment consists of a flow field and obstacles. The flow field data are divided into equidistant grids, with longitude and latitude divisions of 0.5°. A two-dimensional grid coordinate system is constructed with (110° E, 20° N) as the coordinate origin. In order to verify the path planning capability of N-DDQNP proposed in this paper, considering the current environment, planning tasks, and the number of obstacles, the following table was constructed [36]. Experiments were conducted in different current environments, different target points, and different obstacle environments, and the experimental parameters were set as shown in the Table 1.

The C1 results are shown in Table 2. In the E1 environment, the path length of N-DDQNP is 14.91% shorter than that of DQN, and 14.83% less time is saved at the same time. This shows that the exploration mechanism based on a noise network is better than the greedy exploration strategy, which can explore more favorable states to change the value distribution of output actions and maximize the Q-value of the optimal action. In the E2 environment, the obstacle is close to the starting point, which increases the possibility of collision. It is necessary to change the direction of action in advance to prevent collision under the action of the current. At this time, only taking actions consistent with the current will ignore the safety factors. The exploration performance of DDQN is slightly poor [37]. After the same round of training, the length and time of the path generated by DDQN increase by 6.39% and 4.16%, respectively, compared with N-DDQNP. This shows that DDQN did not collect diversified sample information at the beginning of training, resulting in high sample coincidence of the experience pool, and the number of positive samples that the model can use is limited when updating parameters. In the test phase, the greedy strategy is to select the optimal action, and the unknown state will be more difficult to be explored, resulting in the algorithm converging to the local optimal. The E3 and E4 environments are configured with 20 obstacles, which further restricts the feasible range. In comparison with the E1 and E2 environments, the planning time of the I-RRT* algorithm has increased by 1.839 s and 1.669 s, respectively. This suggests that the complexity of the environment has a certain impact on the planning efficiency of the I-RRT* algorithm. In the E3 environment, the N-DDQNP algorithm only requires 3% of the time taken by the I-RRT* algorithm. This demonstrates that the N-DDQNP algorithm satisfies the real-time decision-making requirements for Autonomous Underwater Vehicles (AUVs) during path planning. Generally, the IAPF algorithm can generate the shortest path in specific environments, but its path time is suboptimal due to the more time-consuming process of calculating the gravitational field and repulsive field. The I-RRT* algorithm samples randomly in space and can only generate reachable paths when there are an adequate number of sampling points. In the E4 environment, the path length of the I-RRT* algorithm increases by 20.97% compared to the N-DDQNP model.

**Figure 3:**
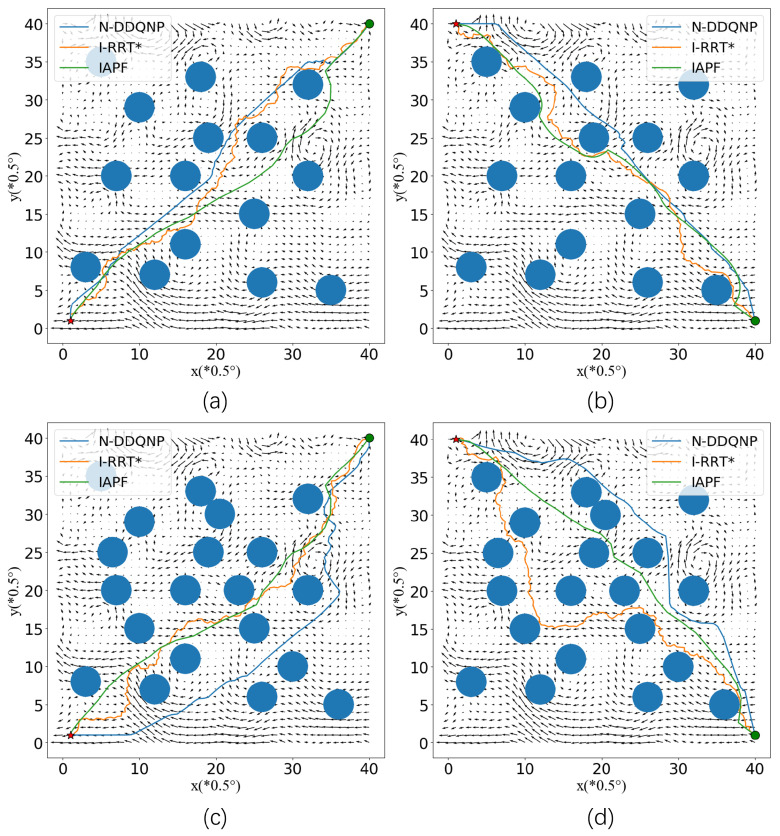
E1–E4 Path. (**a**) Visualization results in E1 Path. (**b**) Visualization results in E2 Path. (**c**) Visualization results in E3 Path. (**d**) Visualization results in E4 Path.

Figure 3 shows the visualization results of paths in the E1–E4 environment. It can be seen that in the E1 environment, the path of I-RRT* is more tortuous [38]. Due to the influence of obstacles, it is necessary to select a feasible point as the next location in the process of generating a random tree. At the same time, under the influence of the current, the direction of the target is opposite to the current. Near the coordinate points (30, 25), the current is swirling. The path of the IAPF algorithm contains a countercurrent area, which increases the path time. At the same time, the path generated by IAPF and I-RRT* algorithm is close to the obstacle, and the position is easily offset in the current environment, resulting in a collision. The N-DDQNP algorithm proposed in this section makes good use of the current near the coordinates (22, 22) and (10, 10), which shortens the overall sailing time. In E2 environment, the path obtained by I-RRT* algorithm runs in the opposite direction to the current at coordinates (10, 35), which will increase AUV travel time. At the same time, for AUV with constant power, countercurrent navigation will increase energy consumption. Under the setting of this target point, there are areas in the space that are consistent with the direction of the AUV target, such as (10, 38). The N-DDQNP algorithm has been fully explored in the training process in combination with the adaptive exploration mechanism of the noise network. AUV can reach the target point faster by taking advantage of the promotion of the ocean current when navigating according to its generated path. In E3 and E4 environments, the number of obstacles increases, which has a greater impact on the path. The reward of collision in N-DDQNP is far less than the negative reward of countercurrent navigation, because the safety of the path is more important than saving time. The N-DDQNP model makes policy adjustments for the environment where obstacles increase and the feasible range decreases, sacrificing a certain path length or time performance in exchange for the absolute security of the path.

In the E1–E4 environment, the path of the I-RRT* algorithm is more tortuous, which requires further smoothing operations in practical applications. The path of the N-DDQNP algorithm is smoother, which reduces unnecessary changes in the speed direction of the AUV during actual navigation. At the same time, it can be clearly seen that the paths generated by I-RRT* and IAPF algorithms are close to obstacles. In the underwater environment disturbed by ocean current, it is necessary to keep a safe distance from obstacles. The path points close to obstacles lead to the decline of path safety. In the process of exploration, N-DDQNP algorithm can not only obtain the location information of the obstacles, but also learn the adverse states around the obstacles. Because the positions around the obstacles have a certain probability of transferring to the collision state, resulting in negative rewards. In order to avoid this situation, N-DDQNP model keeps a certain distance from the obstacles by learning from the samples, which improves the path safety [39].

In the E5–E8 environment, the direction and intensity of the current vary significantly in space. The comparison results of various methods are shown in Table 3. For the E5 environment, the path time of the N-DDQNP planning is 20.65%, 14.53%, 17.90%, and 16.25% shorter than that of the I-RRT*, IAPF, DQN, and DDQN algorithms, respectively. Under the E8 environment, the paths generated by the IAPF and DQN algorithms collide with obstacles at multiple locations, rendering them unsuccessful in reaching the target.

**Figure 4:**
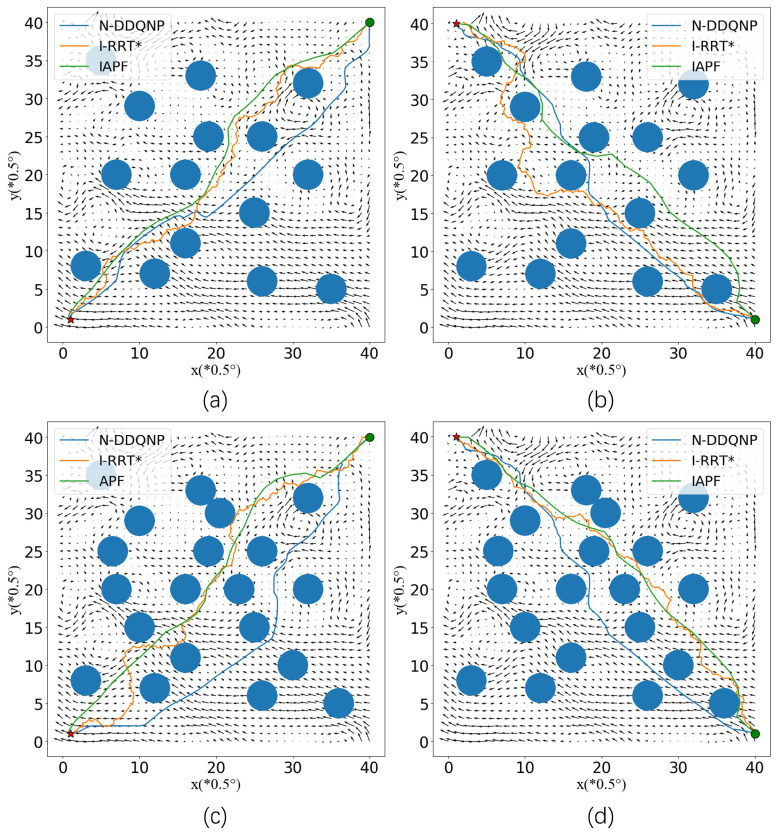
E5–E8 Path. (**a**) Visualization results in E5 Path. (**b**) Visualization results in E6 Path. (**c**) Visualization results in E7 Path. (**d**) Visualization results in E8 Path.

Figure 4 shows the visualization results of paths in the E5–E8 environment. It can be seen that in the E5 environment, the path of I-RRT* is more tortuous. Due to the influence of obstacles, it needs to select a feasible point as the next location in the process of generating a random tree. At the same time, under the influence of the current, the direction of the target is opposite to the current. Near the coordinate points (30, 30), the current is swirling, and the I-RRT* and APF algorithm paths contain countercurrent regions, resulting in an increase in the path time. The N-DDQNP algorithm proposed in this section makes good use of the current near the coordinates (30, 25) and shortens the path time. In the E6 environment, the path obtained by the IAPF algorithm runs in the opposite direction to the current at coordinates (23, 23), which increases the AUV path time. For AUV with constant power, countercurrent navigation will increase energy consumption. Compared with the N-DDQNP algorithm, the noise network is fully explored in the training process.

When it is inevitable to travel against the current, the area with smaller current intensity should be selected as far as possible. In E7 and E8 environments, the number of obstacles increases, which has a greater impact on the path. The reward of collision in N-DDQNP is far less than the negative reward of countercurrent navigation, because the safety of the path is more important than saving time. At the same time, near the (32, 28) area, the AUV passes through the vortex area with the help of the current to reach the target point faster. The N-DDQNP model makes policy adjustments for the environment with increased obstacles and reduced feasible scope. In the E7 environment, compared with the E5 environment, a certain path length or time performance is sacrificed in exchange for the absolute security of the path.

In the E5–E8 environment, the path of the I-RRT* algorithm is more tortuous, which requires further smoothing operations in practical applications. The path of the N-DDQNP algorithm is smoother, which reduces unnecessary changes in the speed direction of the AUV during actual navigation. At the same time, the path of I-RRT* and IAPF has the problem of driving close to obstacles. In the underwater current environment, the path points close to obstacles lead to the decline of path safety. In the E8 environment, although the path generated by IAPF can reach the target, its path collides with the obstacle near the starting point because of the strong driving effect of the current and the gravitational effect of the target point. At the initial stage, N-DDQNP takes the negative x direction as the driving direction, avoiding collision with obstacles near (38, 5).

**Figure 5:**
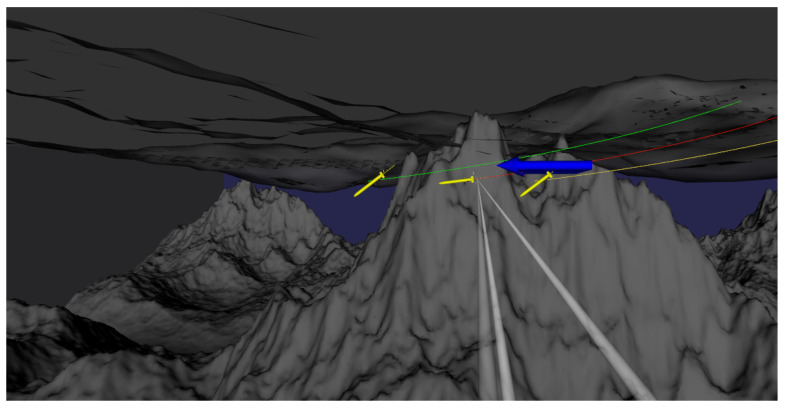
Observation of AUV navigation process through cloud desktop.

Finally, a link between the cloud desktop server and the AUV is constructed, and achieved remote control of the AUV through cloud desktop technology. After each test, the AUV is moved from the ending point to the starting point. At the same time, by using cloud desktop technology to transmit high-quality images, observation and exploration of AUV navigation posture and ocean elements have been achieved on the client side. The AUV travel map during cloud desktop operation is shown in Figure 5.

## 4. Conclusions

This paper presents the application of the noise network in AUV path planning for the first time. To achieve adaptive exploration during the training process and expedite network convergence, an integrated model named N-DDQNP is proposed. Additionally, a dense reward function, which incorporates various factors such as distance, ocean currents, and safety, is defined to generate collision-free paths that optimize time efficiency. The network structure is modified by replacing the original fully connected layer with a noise network. Appropriate noise is introduced to enable random exploration. Furthermore, a reward function with a reference term is designed, and the concept of biased punishment is adopted to enhance the AUV’s utilization of ocean currents. The path planning experiments are conducted in real oceanic current environments to validate the effectiveness of the proposed N-DDQNP algorithm. Results indicate that the path time and security offered by the N-DDQNP algorithm outperform those of traditional IAPF and I-RRT* algorithms. Moreover, the N-DDQNP algorithm exhibits higher convergence speed and average reward when compared to DDQN and DQN models. Finally, the utilization of cloud desktops enables remote device control and facilitates high-quality image transmission. This capability empowers the remote control and observation of AUVs, including monitoring their navigation posture and process. Although the proposed algorithm has demonstrated significant performance improvement, there is still room for enhancement. One possible area for improvement is the training speed of the N-DDQNP algorithm, which could be further accelerated by optimizing sampling efficiency from the perspective of parallel training. Parallel training could involve the construction of multiple sets of intelligent decision-making networks, allowing for the calculation of a total loss function and separate gradient updates. This approach would enable rapid exploration of high-value samples, ultimately enhancing convergence speed.

## Figures and Tables

**Table 1:** Task setting.

		Plan-Task	Obstacle
E1	C1	40,40 → 1,1	10
E2	C1	40,1 → 1,40	10
E3	C1	40,40 → 1,1	15
E4	C1	40,1 → 1,40	15
E5	C2	40,40 → 1,1	10
E6	C2	40,1 → 1,40	10
E7	C2	40,40 → 1,1	15
E8	C2	40,1 → 1,40	15

**Table 2:** C1 Results.

		I-RRT*	IAPF	DQN	DDQN	N-DDQNP
E1	Path Length	370.48	324.79	368.09	336.06	313.21
E1	Path Time	292.32	281.62	300.32	269.17	255.53
E1	Decision Time	6.928	14.783	0.241	0.232	0.219
E2	Path Length	375.72	329.98	353.75	342.62	322.86
E2	Path Time	297.62	279.73	296.92	283.25	271.95
E2	Decision Time	7.379	13.841	0.265	0.253	0.237
E3	Path Length	380.18	332.32	-	389.82	363.24
E3	Path Time	315.62	290.43	-	299.70	285.60
E3	Decision Time	8.767	14.351	-	0.262	0.259
E4	Path Length	421.09	322.75	357.43	332.62	348.97
E4	Path Time	346.49	290.47	305.25	285.60	277.35
E4	Decision Time	9.048	15.732	0.295	0.283	0.279

**Table 3:** C2 Results.

		I-RRT*	IIAPF	DQN	DDQN	N-DDQNP
E5	Path Length	370.85	321.63	363.75	326.62	329.21
E5	Path Time	310.32	288.96	300.75	294.75	246.97
E5	Decision Time	8.267	17.943	0.244	0.242	0.234
E6	Path Length	413.38	330.27	396.75	382.62	328.86
E6	Path Time	393.62	266.73	214.92	288.75	255.95
E6	Decision Time	8.453	18.484	0.269	0.255	0.244
E7	Path Length	406.89	327.23	443.75	352.82	340.66
E7	Path Time	328.62	285.83	299.50	280.50	258.60
E7	Decision Time	8.258	15.851	0.269	0.282	0.265
E8	Path Length	362.87	-	-	342.62	328.97
E8	Path Time	292.28	-	-	274.60	249.35
E8	Decision Time	7.767	-	-	0.263	0.257

## Abbreviations

The following abbreviation are used in this manuscript:

AUV	Autonomous Underwater Vehicle
